# Breast-cancer-specific mortality in patients treated based on the 21-gene assay: a SEER population-based study

**DOI:** 10.1038/npjbcancer.2016.17

**Published:** 2016-06-08

**Authors:** Valentina I Petkov, Dave P Miller, Nadia Howlader, Nathan Gliner, Will Howe, Nicola Schussler, Kathleen Cronin, Frederick L Baehner, Rosemary Cress, Dennis Deapen, Sally L Glaser, Brenda Y Hernandez, Charles F Lynch, Lloyd Mueller, Ann G Schwartz, Stephen M Schwartz, Antoinette Stroup, Carol Sweeney, Thomas C Tucker, Kevin C Ward, Charles Wiggins, Xiao-Cheng Wu, Lynne Penberthy, Steven Shak

**Affiliations:** 1 National Cancer Institute, Bethesda, MD, USA; 2 Genomic Health, Inc., Redwood City, CA, USA; 3 IMS, Inc., Calverton, MD, USA; 4 University of California, San Francisco, CA, USA; 5 Public Health Institute, Cancer Registry of Greater California, Sacramento, CA, USA; 6 University of Southern California, Los Angeles, CA, USA; 7 Cancer Prevention Institute of California, Fremont, CA, USA; 8 Stanford Cancer Institute, Stanford, CA, USA; 9 University of Hawaii Cancer Center, Honolulu, HI, USA; 10 Department of Epidemiology, University of Iowa, Iowa City, IA, USA; 11 Connecticut Tumor Registry, Connecticut Department of Public Health, Hartford, CT, USA; 12 Karmanos Cancer Institute, Wayne State University, Detroit, MI, USA; 13 Cancer Surveillance System, Fred Hutchinson Cancer Research Center, Seattle, WA, USA; 14 Rutgers School of Public Health, Piscataway, NJ, USA; 15 Cancer Institute of New Jersey, New Brunswick, NJ, USA; 16 Utah Cancer Registry, Department of Internal Medicine, and Huntsman Cancer Institute, University of Utah, Salt Lake City, UT, USA; 17 University of Kentucky, Markey Cancer Center, Lexington, KY, USA; 18 Emory University, Atlanta, GA, USA; 19 New Mexico Tumor Registry, University of New Mexico Comprehensive Cancer Center, Albuquerque, NM, USA; 20 Louisiana State University Health Sciences Center, New Orleans, LA, USA

## Abstract

The 21-gene Recurrence Score assay is validated to predict recurrence risk and chemotherapy benefit in hormone-receptor-positive (HR+) invasive breast cancer. To determine prospective breast-cancer-specific mortality (BCSM) outcomes by baseline Recurrence Score results and clinical covariates, the National Cancer Institute collaborated with Genomic Health and 14 population-based registries in the the Surveillance, Epidemiology, and End Results (SEER) Program to electronically supplement cancer surveillance data with Recurrence Score results. The prespecified primary analysis cohort was 40–84 years of age, and had node-negative, HR+, HER2-negative, nonmetastatic disease diagnosed between January 2004 and December 2011 in the entire SEER population, and Recurrence Score results (*N*=38,568). Unadjusted 5-year BCSM were 0.4% (*n*=21,023; 95% confidence interval (CI), 0.3–0.6%), 1.4% (*n*=14,494; 95% CI, 1.1–1.7%), and 4.4% (*n*=3,051; 95% CI, 3.4–5.6%) for Recurrence Score <18, 18–30, and ⩾31 groups, respectively (*P*<0.001). In multivariable analysis adjusted for age, tumor size, grade, and race, the Recurrence Score result predicted BCSM (*P*<0.001). Among patients with node-positive disease (micrometastases and up to three positive nodes; *N*=4,691), 5-year BCSM (unadjusted) was 1.0% (*n*=2,694; 95% CI, 0.5–2.0%), 2.3% (*n*=1,669; 95% CI, 1.3–4.1%), and 14.3% (*n*=328; 95% CI, 8.4–23.8%) for Recurrence Score <18, 18–30, ⩾31 groups, respectively (*P*<0.001). Five-year BCSM by Recurrence Score group are reported for important patient subgroups, including age, race, tumor size, grade, and socioeconomic status. This SEER study represents the largest report of prospective BCSM outcomes based on Recurrence Score results for patients with HR+, HER2-negative, node-negative, or node-positive breast cancer, including subgroups often under-represented in clinical trials.

## Introduction

Despite unprecedented advances in breast cancer diagnosis and treatment, health-care quality and outcomes remain variable, with significant disparities associated with many factors, such as age and race, and the location of care.^1–4^ Leading organizations, including the Institute of Medicine,^5^ the American Society of Clinical Oncology,^6,7^ and the European Organisation for Research and Treatment of Cancer,^8^ have emphasized the need for new research models to more precisely identify what works in clinical practice and encourage appropriate value-based cancer treatment.

In recent years, technologies such as multigene expression analysis, next-generation sequencing, and liquid biopsy, have raised the potential to define appropriate patient subgroups for more precise cost-effective care and improved health outcomes. The 21-gene assay has been clinically validated in ‘prospective–retrospective’ studies on archival tumor tissue to provide both prognostic and predictive information for chemotherapy benefit in early stage, hormone-receptor-positive (HR+), node-negative, or node-positive breast cancer.^9–12^ Recently, the first results from the Trial Assigning Individualized Options for Treatment (TAILORx), a multi-center, prospectively conducted trial of 10,253 women with early-stage breast cancer, were reported.^13^ For the 1,626 trial participants with Recurrence Score results <11 (TAILORx low-range stratum) who received hormonal therapy alone without chemotherapy, 5-year freedom from distant recurrence was 99.3% (95% confidence interval (CI), 98.7–99.6%). These findings demonstrate that patients with low Recurrence Score results can be effectively spared from adjuvant chemotherapy. Results from two other recent studies provide additional evidence of excellent outcomes for patients treated with hormonal therapy alone based on low Recurrence Score results. In the Clalit Health Services study, patients with node-negative or micrometastatic disease and Recurrence Score results <18 (standard low-range group), nearly all of whom (98%) were treated with hormonal therapy without chemotherapy, had <1% risk of distant recurrence and 0% risk of breast-cancer-specific mortality (BCSM) at 5 years.^14^ In the prospective PlanB trial, patients with 0–3 positive lymph nodes and Recurrence Score results ⩽11, who were treated with hormonal therapy alone, had 3-year relapse-free survival exceeding 98%.^15^ As we await results of the TAILORx mid-range stratum (Recurrence Score results 11–25), it would be desirable to have additional evidence of the utility of the 21-gene assay in patients with Recurrence Score result <18, the standard cutoff used in contemporary clinical practice for selection of hormone therapy alone.

Initiated in 1973, the Surveillance, Epidemiology, and End Results (SEER) Program of the National Cancer Institute is an authoritative population-based cancer surveillance program, covering ~30% of the US population and capturing over 98% of incident cancer cases in these covered regions.^16^ SEER Program registries collect standardized patient information on demographics, primary tumor site, and characteristics (histology, grade, stage, and so on), first course of treatment, and survival (survival time, vital status, and cause of death), as mandated by respective state laws. SEER has required the collection of breast cancer multigene test results for cases diagnosed with breast cancer in 2010 and after. To supplement registry data with more complete and accurate multigene test results, we electronically linked Recurrence Score results from the Genomic Health Clinical Laboratory database with each of the SEER registries.

In this first report, we determined the relationship between Recurrence Score results and prospective BCSM for the large population in the SEER program with node-negative and node-positive breast cancer, including subgroups (e.g., racial minorities, the elderly, and the young) that are often under-represented in clinical trials.

## Results

### Population

A total of 379,103 patients were newly diagnosed with primary invasive breast cancer between 2004 and 2011 in the participating SEER registries (Table 1; Supplementary Figure S1). These patients resided in 12 individual SEER states: California (3 registries, 41%), New Jersey (12%), Georgia (11%), Washington (13 Puget Sound region counties only, 6%), Connecticut (5%), Kentucky (5%), Louisiana (5%), Michigan (metropolitan Detroit counties only, 5%), Iowa (4%), Hawaii (2%), New Mexico (2%), and Utah (2%).

Of 379,103 patients, 45,287 (12%) had HR+, nonmetastatic disease and Recurrence Score results, including 40,134 with node-negative disease, 4,691 with micrometastates or up to three positive nodes (N+(mic,1–3)), and 462 with four or more positive nodes or unknown/missing nodal status. Nearly all patients (99.5%) who had 21-gene assay testing had fewer than four positive lymph nodes. Only 165 of 16,202 patients with 4–9 positive nodes and 41 of 7,320 patients with 10 or more positive nodes had testing. Demographic characteristics of tested and untested patients of all ages diagnosed between 2004 and 2011 with node-negative or node-positive (N+(mic,1–3)) are shown in Table 1. Median follow-up of patients with node-negative disease was longer than that of patients with node-positive (N+(mic,1–3)) disease (39 months versus 30 months), reflecting later adoption of the test for use in patients with node-positive disease.

A total of 38,568 patients (10%) were eligible for the prespecified primary analysis (had HR+, HER2-negative, node-negative, nonmetastatic disease; had Recurrence Score results; and were 40–84 years of age; Supplementary Figure S1). Median age of the prespecified primary analysis cohort was 57 years; 99.4% were female; 84% were white, 29% and 54% had tumors of histologic grade 1 and grade 2, respectively; 25% and 53% had tumors of ⩽1 cm and >1 to 2 cm in size, respectively. Median follow-up for the primary analysis cohort was 39 months; 8,239 (21%) patients had >5 years of follow-up.

### Recurrence Score result and breast-cancer-specific mortality (Node-negative)

**Figure 1:**
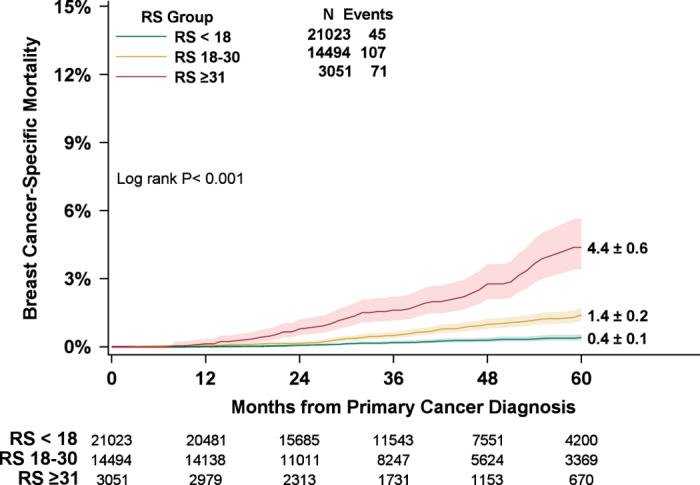
Five-year estimates of breast-cancer-specific mortality, by Recurrence Score group (prespecified primary analysis). Patients with HR+, HER2-negative, node-negative breast cancer who had Recurrence Score (RS) results <18 (green), 18–30 (yellow), or ⩾31 (red) were included in the primary analysis. Five-year estimates of breast-cancer-specific mortality (BCSM) with 95% CIs (green, yellow, and red shading) were 0.4% (0.3–0.6%) in the RS <18 group, 1.4% (1.1–1.7%) in the RS 18–30 group, and 4.4% (3.4–5.6%) in the RS ⩾31 group. Five-year estimates of BCSM±s.e. are shown to the right of their respective lines. Numbers of patients at risk in each group are shown beneath the graph.

Of 38,568 patients in the prespecified primary analysis cohort, 21,023 (55%) had Recurrence Score results <18, 14,494 (38%) had results 18–30, and 3,051 (8%) had results ⩾31. BCSM was significantly associated with Recurrence Score results (*P*<0.001) (Figure 1). Unadjusted 5-year estimates of BCSM were 0.4% (95% CI, 0.3–0.6%), 1.4% (95% CI, 1.1–1.7%), and 4.4% (95% CI, 3.4–5.6%) for patients Recurrence Score results <18, 18–30, and ⩾31, respectively. Chemotherapy use was reported as ‘yes’ in 7%, 34%, and 69% of patients in the Recurrence Score <18, 18–30, and ⩾31 groups, respectively (the remaining patients in each group were reported in SEER as ‘no/unknown chemotherapy use’).

Five-year estimates of overall survival and non-breast-cancer-specific mortality (non-BCSM) were also determined. There was a significant difference in overall survival among Recurrence Score groups (*P*<0.001), with earlier mortality corresponding to Recurrence Score results ⩾31 (data not shown). There was no significant difference in non-BCSM among Recurrence Score groups (*P*=0.10). Five-year other-cause mortality for the primary analysis cohort overall was 5.1% (95% CI, 4.8–5.4%), and 5.1% (95%CI, 4.7–5.6%), 5.0% (95%CI, 4.5–5.5%), and 5.6% (95%CI, 4.6–6.9%) for Recurrence Score <18, 18–30, and ⩾31 groups, respectively. Age was the strongest predictor of non-BCSM (*P*<0.001).

### Recurrence Score result and breast-cancer-specific mortality (Node-positive)

A total of 4,691 patients with positive lymph nodes (N+(mic,1–3)) had Recurrence Score results (Supplementary Figure S1). Of these, 2,694 (57%) had Recurrence Score results <18, 1,669 (36%) had results 18–30, and 328 (7%) had results ⩾31. In addition, 50% of these patients had tumors >10 to 20 mm in size, 55% had moderate grade tumors, and 78.3% were 50 years of age or older (Table 1). Among patients with node-positive (N+(mic,1–3)) disease, 5-year BCSM was significantly different for the three Recurrence Score groups: 1.0% (95% CI, 0.5–2.0%) in the <18 group, 2.3% (95% CI, 1.3–4.1%) in the 18–30 group, and 14.3% (95% CI, 8.4–23.8%) in the ⩾31 group (*P*<0.001). Chemotherapy use was reported as ‘yes’ (rather than ‘no/unknown’) in 23%, 47%, and 75% of patients in the Recurrence Score <18, 18–30, and ⩾31 groups, respectively. Excluding patients with micrometastatic disease, the 5-year BCSM for patients with Recurrence Score results <18 and 1–3 positive nodes (*n*=2,617) was 1.3% (95% CI, 0.6–2.9%).

### Subgroups and breast-cancer-specific mortality

**Figure 2:**
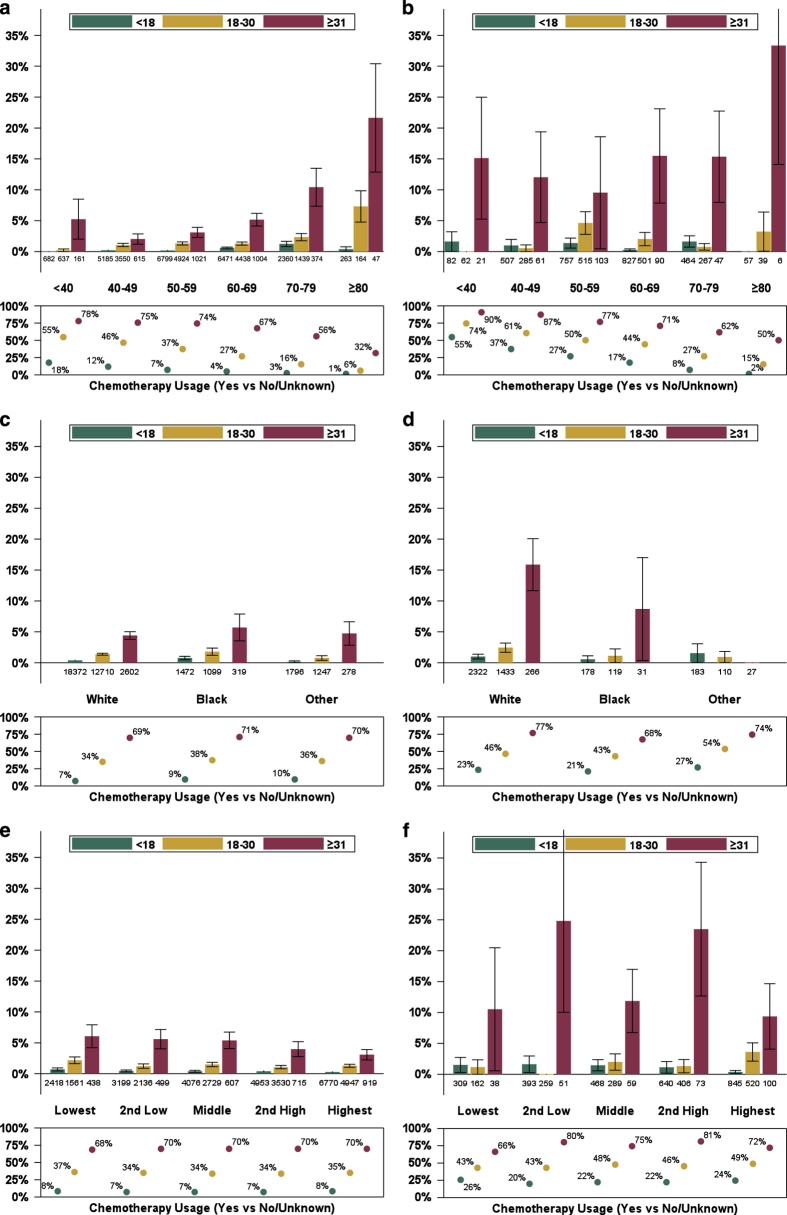

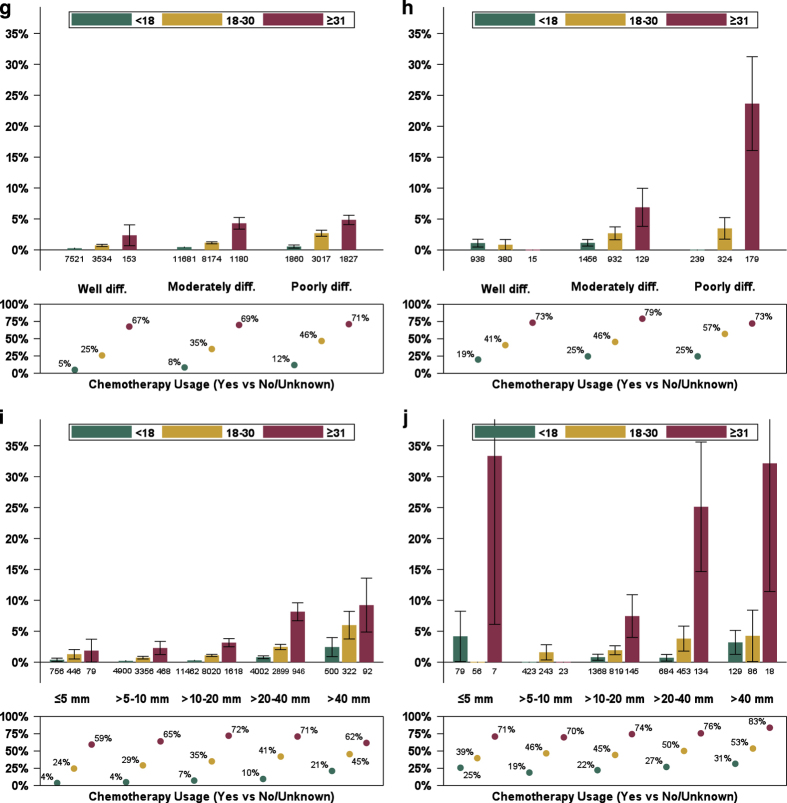
Five-year estimates of breast cancer-specific mortality, by Recurrence Score group (subgroup analyses). Patients with HR+, HER2-positive, node-negative (**a**,**c**,**e**,**g**,**i**) and node-positive (micrometastases up to three positive nodes; **b,d,f,h,j**) breast cancer who had Recurrence Score (RS) results <18 (green), 18–30 (yellow), or ⩾31 (red) were included in subgroup analyses by age (**a**,**b**), race (**c**,**d**), socioeconomic status (**e**,**f**) as defined by the Yost composite index,^41^ tumor grade (**g**,**h**), and tumor size (**i**,**j**). Five-year estimates of breast-cancer-specific mortality (BCSM)±s.e. are shown. Percentages of patients with chemotherapy use reported as ‘yes’ as a proportion of all patients (‘yes’ or ‘no/unknown’ chemotherapy use) are shown beneath the graph.

Recurrence Score group was significantly prognostic (*P*⩽0.001) for 5-year BCSM for the node-negative and the node-positive (N+(mic,1–3)) populations as wholes, in every node-negative subgroup of age, grade, race, and socioeconomic (SES) status, and in the node-positive (N+(mic,1–3)) subgroups with substantial numbers of patients (>10 to 20 mm, moderate grade, age 60–69 years, and White; Figure 2a–j Supplementary Table S1). Notably, 5-year BCSM was 1.3% or lower for patients with Recurrence Score results <18, regardless of nodal status and age group. Similarly, low BCSM was observed for patients <70 years of age with Recurrence Score results 18–30. However, for patients with Recurrence Score results ⩾31, 5-year BCSM was substantially higher and ranged from 2.0 to 21.6%, depending on age group (Figure 2a). Among patients with node-positive (N+(mic,1–3)) disease, those with Recurrence Score results ⩾31 had 5-year BCSM that exceeded 9.5%, regardless of age group (Figure 2b). For patients with both node-negative and node-positive (N+(mic,1–3)) disease, reported chemotherapy use generally decreased with increasing age.

For patients with node-negative or node-positive (N+(mic,1–3)) disease, those with higher Recurrence Score results had higher 5-year BCSM than those with lower results, regardless of race (White or Black; Figure 2c,d) and regardless of SES quintile (Figure 2e,f). Of note, among patients with node-negative disease and Recurrence Score results ⩾31, 5-year BCSM appeared to decrease with higher quintiles, despite similar reported chemotherapy use across SES quintiles (68–70%; Figure 2e). Among patients with node-positive (N+(mic,1–3)) disease and Recurrence Score results ⩾31, 5-year BCSM exceeded 9%, regardless of SES quintile (Figure 2f).

Among patients with node-negative disease, Recurrence Score group was significantly prognostic (*P*<0.05) for subgroups analyzed by tumor grade (Figure 2g) and tumor size (Figure 2i), except for patients with very small tumors. For patients with tumors ⩽5 mm in size, the estimated risk is elevated in the group with Recurrence Score results ⩾31, although the estimate lacks precision (1.9%; 95% CI, 0.3–12.5%). Among patients with node-negative disease and tumors >4 cm in size, Recurrence Score group was significantly prognostic (*P*=0.005), but all three Recurrence Score groups have estimated 5-year BCSM of 2.4% or higher (Figure 2i). For patients with node-positive (N+(mic,1–3)) disease, those with higher Recurrence Score results generally had worse 5-year BCSM than those with lower results, regardless of tumor size (Figure 2j).

With respect to tumor grade, regardless of nodal status, 5-year BCSM generally increased with worsening grade, as expected (Figure 2g,h). Within grade categories, however, the group with Recurrence Score results <18 consistently had low 5-year BCSM (<1% for node-negative disease; <2% for node-positive (N+(mic,1–3)) disease), even for patients with high tumor grade. For patients with node-negative disease and Recurrence Score results ⩾31, 5-year BCSM was >2%, even for patients with low tumor grade.

### Multivariable model with adjustment for baseline covariates

The effects of Recurrence Score results and potential confounding variables were assessed using a Cox regression model. Compared with patients with a Recurrence Score result <18 and without adjusting for other covariates, patients with Recurrence Score results 18–30 and results ⩾31 had higher hazards for BCSM. These effects were modestly attenuated after adjustment for grade, tumor size, age, and race (Table 2); however, the Recurrence Score 18–30 and ⩾31 groups remain at significantly increased hazards (*P*<0.001). Because Recurrence Score results are known to affect treatment decisions, an additional model was fit with a treatment interaction. In this model, Recurrence Score results remained prognostic among both patients for whom chemotherapy was reported as ‘yes’ and as ‘no/unknown,’ but the strength of the association was attenuated for those with chemotherapy reported as ‘yes’ (*P*=0.03 for covariate-adjusted interaction). Comparable models fitted using Recurrence Score result as a continuous linear variable were also significant for prognosis, with and without adjustment for covariates (*P*<0.001 for both).

In the covariate-adjusted model, increases in the hazard of BCSM were significantly associated with poorly differentiated tumors (hazard ratio (HR) 2.1 (95% CI, 1.3–3.2), *P*=0.002), tumors >4 cm in size (HR 3.4 (95% CI, 1.3–8.7), *P*=0.010), age 70–79 years (HR 2.4 (95% CI, 1.2–5.0), *P*=0.014), and age ⩾80 years (HR 6.1 (95% CI, 2.6–14.3), *P*<0.001). Patients with very small tumors (⩽5 mm) did not have significantly different outcomes than patients had with tumors >5 to 10 mm in size. The youngest patients (<40 years) did not have significantly different outcomes than patients who were 40–49 years or 50–59 years of age. In contrast, older patients at the time of diagnosis (⩾70 years) had significantly worse BCSM in both the unadjusted and adjusted models.

The multivariable model also demonstrated differences in outcomes by race. An alternate model, replacing race with Yost quintile, as a marker for neighborhood SES, also showed worsening outcomes with lower SES; however, neither race nor SES were significant when both terms were included in the same model, and both effects are relatively weak compared to the genomic and clinical factors.

### TAILORx clinical trial cutpoints

Since its initial development, the 21-gene assay results report has used Recurrence Score cutpoints of 18 and 31, and has provided individualized risk estimates for the specific Recurrence Score result for each patient. Alternative cutpoints, however, have been implemented in clinical trials. The ongoing TAILORx randomized trial uses cutpoints of 11 and 25 to define Recurrence Score categories <11, 11–25, and >25.^13^ Applying these alternative categories to our study cohort, the 5-year BCSM in the HR+, node-negative, non-age-restricted cohort were 0.4% (95% CI, 0.2–0.6%) for 7,281 patients with Recurrence Score results <11, 0.7% (95% CI, 0.6–0.8%) for 26,462 patients with Recurrence Score results 11–25, and 3.6% (95% CI, 3.0–4.4%) for 6,391 patients with Recurrence Score results >25 (*P*<0.001). Considering both the standard cutpoints and the TAILORx cutpoints, the 5-year BCSM for 10,589 patients with Recurrence Score results 18–24 and 3,905 patients with Recurrence Score results 25–30 were 1.0% (95% CI, 0.8–1.4%) and 2.4% (95% CI, 1.8–3.2%), respectively.

## Discussion

Our prespecified primary analysis of the population-based SEER database electronically supplemented with Recurrence Score results showed that the Recurrence Score result was significantly associated with the likelihood of BCSM (*P*<0.001). In multivariable analysis that adjusted for the prognostic baseline covariates of patient age, tumor size, tumor grade, and race, as well as reported chemotherapy use, the Recurrence Score result remained strongly predictive of BCSM (*P*<0.001).

We showed that patients with node-negative disease in the SEER program with Recurrence Score results <18, who had low rates of chemotherapy use reported as ‘yes’ (7%), had 5-year BCSM of 0.4% (95% CI, 0.3–0.6%), a finding that is consistent with those of earlier, prospective–retrospective clinical validation studies performed on archival specimens.^9,11,17^ For example, 5-year BCSM for patients with Recurrence Score results <18 was 0.9% (95% CI, 0.3–2.8%) in the National Surgical Adjuvant Breast and Bowel Project (NSABP) B-14 study and 1.1% (95% CI, 0.5–1.6%) in the Kaiser study (L. Habel, personal communication). That our results for patients with Recurrence Score results <18 were similar to earlier results, despite dissimilar time periods in which each cohort was enrolled, demonstrates the capacity of the Recurrence Score result to identify patients with excellent prognosis, regardless of the era in which they were diagnosed or the specific treatments they received. Moreover, the favorable outcomes we observed in patients with node-negative disease and Recurrence Score results <18 confirm—and extend—findings of the TAILORx trial, the Clalit Health Services study, and the PlanB trial of the Women’s Healthcare Study Group in Germany, all of which reported excellent outcomes for patients with low Recurrence Score results.^13–15^

For patients with node-positive (N+(mic,1–3)) disease, the Recurrence Score result was strongly predictive of BCSM, despite the relatively short-median follow-up. For patients with Recurrence Score results <18, 23% of whom had chemotherapy use reported as ‘yes,’ the 5-year BCSM was 1.0% (95% CI, 0.5–2.0%), similar to the 5-year BCSM observed for patients with node-negative disease. These results in more than 4,600 patients with node-positive disease reconfirm the Recurrence Score results from the SWOG 8814 and ATAC studies showing that the 21-gene assay identifies a group of patients with node-positive disease and Recurrence Score results <18 with favorable outcomes.^11,12^ The ongoing Treatment (Rx) for Positive Node, Endocrine Responsive Breast Cancer (RxPONDER) clinical trial,^18^ in which patients with node-positive disease and Recurrence Score results <25 are randomized to endocrine therapy with or without adjuvant chemotherapy, should provide more definitive information on the effect of chemotherapy. Our SEER results provide prospective evidence that certain patients with node-positive disease and low Recurrence Score results have favorable 5-year BCSM, and reassurance that the randomization in RxPONDER was justified.

Prior to our study, there were relatively little data regarding Recurrence Score results and outcomes in younger and older patients with breast cancer. For example, only 3% of patients in the NSABP B-14 study,^19^ 5% in the ongoing TAILORx trial (J. Sparano, personal communication), and 0% in the ongoing MINDACT trial were older than 70 years.^20^ Our population-based SEER study is therefore noteworthy for providing the largest experience to date with patients ⩾70 years of age, including 4,647 with node-negative disease and 880 with node-positive (N+(mic,1–3)) disease, and with patients <40 years, including 1,480 with node-negative disease and 165 with node-positive (N+(mic,1–3)) disease. Our study found that regardless of age, patients with Recurrence Score results <18 had excellent outcomes: 5-year BCSM was <1.3% in the node-negative group and <1.7% in the node-positive (N+(mic,1–3)) group. Importantly, although younger age is considered an unfavorable prognostic factor,^21^ the 5-year BCSM of 0.0% that we observed in 682 patients <40 years with node-negative disease and Recurrence Score results <18 indicates that the Recurrence Score assay identified a subset of very young patients that had excellent outcomes.

We noted that outcomes were especially poor for older patients with node-negative disease and Recurrence Score results ⩾31: 5-year BCSM was 10.4% for patients 70–79 years and 21.6% for patients ⩾80 years. Within this Recurrence Score group (⩾31), 72% of patients <70 years, but only 53% of patients ⩾70 years, had chemotherapy use reported as ‘yes.’ Previous studies have reported that older women with breast cancer generally receive less aggressive treatment than younger patients do and are more likely to die from their disease.^22–25^ Importantly, practice guidelines by the National Comprehensive Cancer Network on ‘Older Adult Oncology’ acknowledge that older women with breast cancer ‘often do not receive ‘standard of care.^26^’ Clearly, as population demographics shift, it is imperative that we understand and take action to lessen BCSM in older patients. A more-detailed analysis of the older population in SEER is underway to examine comorbidities, competing risks, and outcomes in patients with and without 21-gene assay testing.

We further show that the Recurrence Score result was significantly prognostic for BCSM in every subgroup of race, SES, and pathology (with the possible exception of tumors ⩽5 mm in size, although the general trend for this subgroup was consistent with observations made in subgroups with larger tumors). For several subgroups historically under-represented in clinical studies, including patients of lower SES and patients who identify as non-White, our results add substantially to the body of knowledge relating the Recurrence Score results to outcomes.

Finally, Recurrence Score biology at diagnosis was a very strong predictor of BCSM but, as expected, was not predictive of other-cause mortality. Age was the strongest predictor of other-cause mortality. It will be important in future studies to assess in greater detail BCSM in the context of other-cause mortality and patient comorbidities.

The availability now of prospective outcomes for over 50,000 patients with Recurrence Score results across multiple studies carries implications for both clinical practice and breast cancer staging. Use of clinical and pathologic factors alone (e.g., luminal A-like or luminal B-like) may be inadequate to select patients for consideration of adjuvant chemotherapy. The 21-gene Recurrence Score assay has the capacity to further risk-stratify within any clinical or pathologic category (including luminal A-like and luminal B-like),^27,28^ based on tumor biology. At present, the American Joint Committee on Cancer (AJCC) is working to revise the current TNM breast cancer staging system, largely limited to anatomic information, to incorporate new molecular testing information. Collaborations between SEER and diagnostic testing companies, such as the research model pioneered in this study, can provide evidence from large high-quality datasets to support updated criteria for cancer staging.

In general, observational studies can provide valuable information on diagnosis, treatment, and outcomes in actual clinical practice. When very large, observational studies can provide new information about subgroups of patients that randomized clinical trials are often not powered to assess. Nonetheless, observational studies, like ours, have limitations. First, data in the SEER Program are derived from patients who were not randomized to treatment. This potentially introduces bias and confounding factors for estimating treatment effects that cannot be fully controlled. To enhance the rigor of our large observational study, we applied the Good Research for Comparative Effectiveness (GRACE) Principles of good study design and implementation to the methodology and analyses.^29^ Second, the SEER Program collects no information on breast cancer recurrence and progression. Third, chemotherapy use is under-reported in SEER. Although the magnitude of under-reporting is unknown, one study involving the Medicare population suggested a 30% relative under-ascertainment.^30^ For the reasons of under-reporting of chemotherapy use and selection bias in who elected to get chemotherapy, we do not report BCSM by treatment choice. Although we do report the percent for whom chemotherapy is reported as ‘yes’ versus ‘no/unknown’ in various subgroups, the limitations of this variable should be kept in mind when interpreting these results. At the time of this analysis with SEER survival follow-up only through 2012, the extent of patient follow-up beyond 5 years was limited. However, with the large sample size, the confidence intervals for the 5-year BCSM estimates are narrow. Moreover, the reported overall benefit of chemotherapy has been observed by 5 years.^31^ Finally, other multigene signatures for breast recurrence risk were not included in this analysis. It should be noted that the 21-gene assay accounted for >93% of tests reported to SEER by manual collection in 2010–2012.

Our study nevertheless had a number of strengths. First, ours is the largest-to-date study of prospective outcomes based on Recurrence Score results in node-negative and node-positive disease. Second, the SEER Program is remarkable for its stringent ascertainment of population-based patient-specific data at the time of diagnosis and at the time of death. Third, the population-based nature of the SEER registries combined with the size of the database of the Genomic Health Clinical Laboratory (the only laboratory that performs the 21-gene assay) ensures that study results reflect real-world practices.

In the future, we plan to conduct additional analyses, including among others: (a) continued follow-up of the SEER registries to encompass an ever-increasing number of patients; (b) in-depth analyses of important subgroups by, for example, race, ethnicity, SES, and sex; (c) determination of propensity scores for chemotherapy benefit; (d) assessment of factors that influence assay ordering, including geography; and (e) comparison of manually collected and Genomic Health Clinical Laboratory-reported Recurrence Score results. In addition, we plan to conduct an analogous analysis after merging with the SEER-Medicare database.

In conclusion, this study represents a new model for collaboration between National Cancer Institute, SEER registries, and industry to more efficiently and completely capture important genomic test results to inform understanding of ‘real-world’ oncology practice. Our study results strongly reinforce the findings of the prospectively designed TAILORx trial, other prospective outcomes studies, and numerous earlier prospective–retrospective validation studies.^9–15,17^ Our SEER study provides additional evidence in >44,000 patients with node-negative and node-positive (N+(mic,1–3)) disease that the 21-gene assay accurately predicts prospective outcomes, independent of patient age, tumor size, and tumor grade.

## Materials and methods

### Study population

The electronic linkage of the 21-gene Oncotype DX Breast Recurrence Score assay (Genomic Health, Inc., Redwood City, CA, USA) results from the Genomic Health Clinical Laboratory database with the SEER registries database was based on protected health information included in the these databases. The linkage was performed by Information Management Services (IMS, Calverton, MD, USA), a National Cancer Institute contractor that manages cancer surveillance data for SEER Program registries, using the Link Plus software (Centers for Disease Control and Prevention, Division of Cancer Prevention and Control, National Program of Cancer Registries; Atlanta, GA, USA), a deterministic SAS algorithm, and manual adjudication of partial matches by registries staff. De-identified data were released to the study team after SEER approval of a custom data request. This linkage allowed for the inclusion of Recurrence Score results from 2004 and for more complete Recurrence Score capture from 2010 forward (manuscript in preparation).

The primary survival analysis cohort, statistical methodology, and study end point were prespecified before the data linkage was performed. The primary survival analysis cohort was specified as all patients in the SEER research database diagnosed between 1 January 2004 and 31 December 2011 who had lymph node-negative, HR+, HER2-negative, primary invasive breast cancer, and a Recurrence Score result in the Genomic Health Clinical Laboratory database. Standard cutoffs that define risk groups by Recurrence Score results are as follows: low (<18), intermediate (18–30), and high (⩾31). Patients were excluded if they had metastatic disease, any previous history of invasive cancer (but not prior ductal carcinoma *in situ*), or multiple Recurrence Score results for any reason (e.g., multifocal disease or concurrent primary tumors).

To be more consistent with the patient populations of the NSABP B-14 and Kaiser clinical validation studies,^9,17^ the primary survival analysis was restricted to patients with lymph node-negative, HR+ HER2-negative breast cancer who were 40–84 years of age at diagnosis. Secondary survival analyses of various subgroups included patients of all ages and patients with node-positive disease.

Patients were considered HR+ if their tumors were estrogen receptor- or progesterone receptor-positive (ER+ or PR+) by the SEER-reported ER and PR immunohistochemistry results (borderline results were considered positive) and by the 21-gene assay quantitative reverse transcription-PCR (RT-PCR) single-gene ER or PR results. Patients were considered node-negative or node-positive based on the data collected by SEER. According to the AJCC 6th and 7th edition,^32^ micrometastases were considered lymph node-positive and isolated tumor cells were considered lymph node-negative. The SEER database includes no information on HER2 status prior to 2010. Thus, HER2-negative patients were identified among those with Recurrence Score results who had 21-gene assay quantitative RT-PCR single-gene HER2 scores ⩽11.5.^33–35^ Rare cases in which tumors were classified in SEER as undifferentiated or anaplastic were considered to be poorly differentiated. Both male and female patients were included in all analyses.

### End point

The study end point for all outcome analyses was BCSM. Underlying causes of death (CODs) were ascertained by the SEER registries through linkages with state death certificates and the National Death Index from the National Center for Health Statistics.^36^ In addition, vital status was ascertained through linkages with other sources, such as state Departments of Motor Vehicles, state voter registration databases, and the Social Security Administration. To correct the known errors with COD attribution, the SEER program developed a special COD variable that maps underlying CODs to the primary cancer diagnosis.^37^ We used this variable to assign a broad set of CODs to capture deaths from breast cancer among patients diagnosed with an incident breast cancer in SEER. Patients who did not die of breast cancer were censored at time of last follow-up (31 December 2011) or at time of death from other causes.

To protect patient confidentiality, time to event in SEER is captured in months rather than days. Therefore, actuarial methods rather than Kaplan–Meier methods were utilized for event and freedom-from-event calculations. Five-year BCSM was selected as the summary outcome measure of greatest importance based on its importance as a clinically meaningful end point and limited BCSM follow-up beyond 5 years.

### Statistical analysis

The primary survival analysis was prespecified to be performed on eligible patients in the three categorical Recurrence Score groups based on the standard 21-gene assay Recurrence Score cutpoints: <18, 18–30, or ⩾31. The continuous Recurrence Score result (0–100) was evaluated in secondary analyses. Actuarial estimates of survival were computed through 5 years with 95% CIs. The log-rank test was used to compare the three Recurrence Score groups. Hazard ratios were calculated using Cox regression models, which were fit using SAS PROC PHREG (SAS/STAT version 9.4, SAS Institute Inc., Cary, NC, USA). Proportional hazards assumptions were assessed and met in the final models. Two sets of prognostic models were fit: one set used the three-category Recurrence Score group variable and the second, as a sensitivity analysis, used the continuous Recurrence Score result.

It is important to note that the Recurrence Score result is provided to patients and physicians to guide treatment decisions. Although the result is one of many factors that influence treatment decision-making, the group with Recurrence Score results <18 has been shown in multiple previous studies to have a much lower rate of reported chemotherapy use than the groups with higher Recurrence Score results.^38–41^ Thus, the difference in BCSM curves among the three Recurrence Score groups was expected to be smaller, in percentage terms, than if all patients were treated with hormonal therapy alone. Adjusting for treatment was not straightforward because treatment itself is influenced by the Recurrence Score result and there is a known issue with ascertainment bias for treatment. As a sensitivity analysis, multivariable models were fit including Recurrence Score results, treatment, and an interaction in the model; however, these models should not be used to evaluate comparative effectiveness.

Subgroup analyses were conducted by age, race, tumor size, tumor grade, and SES Index. The SES Index was created by SEER and is based on each patient’s census-tract at-diagnosis attributes reflected by the Yost composite Index. To further protect patients’ privacy, the SES Index is categorized in quintiles.^42^

## Figures and Tables

**Table 1:** Patient demographics, by nodal status and Recurrence Score assay status (*N*=241,681)

*Category*	*Parameter*	*Node-negative, HR+ (*N*=184,190)*		*Node-positive (*N*+(mic,1-3))*^a^*, HR+ (*N*=57,491)*	
		*HER2− With RS (*N*=40,134)*	*HER2±*^b^ *Without RS (*N*=144,056)*	*HER2− With RS (*N*=4,691)*	*HER2±*^b^ *Without RS (*N*=52,800)*
		n *(%)*	n *(%)*	n *(%)*	n *(%)*
Age	<40 years	1,480 (3.7)	4,827 (3.4)	165 (3.5)	3,889 (7.4)
	40–49 years	9,350 (23.3)	21,492 (14.9)	853 (18.2)	12,513 (23.7)
	50–59 years	12,744 (31.8)	31,230 (21.7)	1,375 (29.3)	14,032 (26.6)
	60–69 years	11,913 (29.7)	35,676 (24.8)	1,418 (30.2)	11,536 (21.8)
	70–79 years	4,173 (10.4)	30,548 (21.2)	778 (16.6)	6,952 (13.2)
	⩾80 years	474 (1.2)	20,283 (14.1)	102 (2.2)	3,878 (7.3)
					
Sex	Male	235 (0.6)	999 (0.7)	34 (0.7)	559 (1.1)
	Female	39,899 (99.4)	143,057 (99.3)	4,657 (99.3)	52,241 (98.9)
					
Race	White	33,684 (83.9)	119,790 (83.2)	4,021 (85.7)	42,723 (80.9)
	Black	2,890 (7.2)	11,539 (8.0)	328 (7.0)	5,357 (10.1)
	Other	3,321 (8.3)	11,940 (8.3)	320 (6.8)	4,434 (8.4)
	Unknown	239 (0.6)	787 (0.5)	22 (0.5)	286 (0.5)
					
Tumor size^c^	⩽5 mm	1,281 (3.2)	20,601 (14.6)	142 (3.0)	1,393 (2.7)
	>5–10 mm	8,724 (21.9)	36,005 (25.5)	689 (14.8)	4,339 (8.4)
	>10–20 mm	21,100 (52.9)	51,530 (36.5)	2,332 (50.0)	19,036 (36.7)
	>20–40 mm	7,847 (19.7)	26,521 (18.8)	1,271 (27.2)	20,078 (38.7)
	>40 mm	914 (2.3)	6,465 (4.6)	233 (5.0)	7,030 (13.6)
					
Tumor grade^c^	Well	11,208 (28.8)	44,742 (32.9)	1,333 (29.0)	8,525 (16.8)
	Moderate	21,035 (54.0)	62,320 (45.8)	2,517 (54.8)	25,334 (50.1)
	Poor^d^	6,704 (17.2)	28,888 (21.2)	742 (16.2)	16,742 (33.1)
					
Reported CT use	No/unknown	31,023 (77.3)	112,008 (77.8)	3,048 (65.0)	17,258 (32.7)
	Yes	9,111 (22.7)	32,048 (22.2)	1,643 (35.0)	35,542 (67.3)

Abbreviations: CT, chemotherapy; HER2, human epidermal growth factor receptor 2; HR, hormone receptor; RS, Recurrence Score result.

a Excludes patients with ⩾4 positive nodes or unknown/missing nodal status.

b Excludes patients with HER2-positive breast cancer in cohorts with Recurrence Score results, based on 21-gene assay quantitative single-gene HER2 result (by reverse transcription-PCR); includes patients with HER2-positive breast cancer in cohorts without Recurrence Score results because HER2 status was not reported to SEER before 2010.

c Among patients in the cohort with nonmissing information.

d Includes undifferentiated and anaplastic tumors.

**Table 2:** Multivariable model for breast-cancer-specific mortality (patients with hormone-receptor-positive, HER2-negative, node-negative breast cancer, *N*=40,134)

*Characteristic*	*Effect*	*Unadjusted HR (95% CI)*	*Unadjusted* P *value*^a^	*Adjusted HR (95% CI)*	*Adjusted* P *value*^a^
RS group (versus RS <18)	RS 18–30	3.1 (2.3, 4.3)	<0.001	3.0 (2.1, 4.2)	<0.001
	RS ⩾31	11.0 (7.8, 15.5)		7.8 (5.3, 11.6)	
					
Tumor grade (versus well-differentiated)	Moderately differentiated	2.2 (1.5, 3.4)	<0.001	1.6 (1.0, 2.4)	0.005
	Poorly differentiated^b^	5.5 (3.7, 8.4)		2.1 (1.3, 3.2)	
					
Tumor size (versus ⩽5 mm)	>5 to 10 mm	0.6 (0.3, 1.4)	<0.001	0.7 (0.3, 1.6)	<0.001
	>10 to 20 mm	0.9 (0.4, 2.0)		0.9 (0.4, 2.1)	
	>20 to 40 mm	2.5 (1.2, 5.4)		1.9 (0.8, 4.3)	
	>40 mm	4.5 (1.9, 10.7)		3.4 (1.3, 8.7)	
					
Age (versus <40 years)	40–49	0.7 (0.3, 1.4)	<0.001	0.8 (0.4, 1.7)	<0.001
	50–59	0.8 (0.4, 1.6)		0.9 (0.5, 1.9)	
	60–69	1.3 (0.7, 2.7)		1.4 (0.7, 2.8)	
	70–79	2.6 (1.3, 5.3)		2.4 (1.2, 5.0)	
	80–89	7.3 (3.2, 16.7)		6.1 (2.6, 14.3)	
					
Race (versus White)	Black	1.9 (1.3, 2.7)	0.008	1.7 (1.1, 2.5)	0.036
	Other	0.9 (0.5, 1.4)		0.9 (0.5, 1.5)	

Abbreviations: CI, confidence interval; CT, chemotherapy; HR, hazard ratio; RS, Recurrence Score result.

a Likelihood ratio *P* value.

b Includes undifferentiated and anaplastic tumors.
