# Author Correction: Breast-cancer-specific mortality in patients treated based on the 21-gene assay: a SEER population-based study

**DOI:** 10.1038/s41523-018-0069-3

**Published:** 2018-07-06

**Authors:** Valentina I Petkov, Dave P Miller, Nadia Howlader, Nathan Gliner, Will Howe, Nicola Schussler, Kathleen Cronin, Frederick L Baehner, Rosemary Cress, Dennis Deapen, Sally L Glaser, Brenda Y Hernandez, Charles F Lynch, Lloyd Mueller, Ann G Schwartz, Stephen M Schwartz, Antoinette Stroup, Carol Sweeney, Thomas C Tucker, Kevin C Ward, Charles Wiggins, Xiao-Cheng Wu, Lynne Penberthy, Steven Shak

**Affiliations:** 1 National Cancer Institute, Bethesda, MD USA; 2 Genomic Health, Inc., Redwood City, CA USA; 3 IMS, Inc., Calverton, MD USA; 4 University of California, San Francisco, CA USA; 5 Public Health Institute, Cancer Registry of Greater California, Sacramento, CA USA; 6 University of Southern California, Los Angeles, CA USA; 7 Cancer Prevention Institute of California, Fremont, CA USA; 8 Stanford Cancer Institute, Stanford, CA USA; 9 University of Hawaii Cancer Center, Honolulu, HI USA; 10 Department of Epidemiology, University of Iowa, Iowa City, IA USA; 11 Connecticut Tumor Registry, Connecticut Department of Public Health, Hartford, CT USA; 12 Karmanos Cancer Institute, Wayne State University, Detroit, MI USA; 13 Cancer Surveillance System, Fred Hutchinson Cancer Research Center, Seattle, WA USA; 14 Rutgers School of Public Health, Piscataway, NJ USA; 15 Cancer Institute of New Jersey, New Brunswick, NJ USA; 16 Utah Cancer Registry, Department of Internal Medicine, and Huntsman Cancer Institute, University of Utah, Salt Lake City, UT USA; 17 University of Kentucky, Markey Cancer Center, Lexington, KY USA; 18 Emory University, Atlanta, GA USA; 19 New Mexico Tumor Registry, University of New Mexico Comprehensive Cancer Center, Albuquerque, NM USA; 20 Louisiana State University Health Sciences Center, New Orleans, LA USA

**Correction to**: *npj Breast Cancer*; 10.1038/npjbcancer.2016.17, Published online 8 June 2016

In the original version of the published article, line three of the third paragraph of the methods stated “Excluding patients with micrometastatic disease, the 5-year BCSM for patients with Recurrence Score results <18 and 1–3 positive nodes (*n* = 2,617) was 1.3% (95% CI, 0.6–2.9%).” To improve clarity this statement has been replaced with “Excluding patients with micrometastatic disease, there were 2,617 patients with 1–3 positive nodes. Of these, 1,487 also had Recurrence Score results <18 with 5-year BCSM of 1.3% (95% CI, 0.6–2.9%).” The original version of the published article also contained an error in the second sentence of the Figure 2 legend describing the mutation status of the patient population examined. The sentence in the original published version of the Article stated “Patients with HR+, HER2-positive, node-negative…” this has been changed to “Patients with HR+, HER2-negative, node-negative…”. This has been corrected in the PDF and HTML versions of this paper.

