# Supplementary material for: Breast-cancer-specific mortality in patients treated based on the 21-gene assay: a SEER population-based study
Source: NPJ Breast Cancer. 2016 Jun 8;2:16017–. doi: 10.1038/npjbcancer.2016.17 (PMC5515329; doi:10.1038/npjbcancer.2016.17)
Supplement: Supplementary Figure Legend [file npjbcancer201617-s1.doc]

**Supplemental Figure 1. STROBE diagram.** The prespecified primary analysis cohort is shown in gray. ^1^Patients with ≥4 positive nodes (n=23,522) or with unknown/missing nodal status (n=7,727) are not included. ^2^Patients were also HER2-negative by 21-gene assay single-gene HER2 result (by reverse transcription polymerase chain reaction). ^3^Of 45,287 patients with Recurrence score results, 5,153 had node-positive disease; 4,691 with [N+(mic,1-3)] are included in the analyses, but those with ≥4 positive nodes (n=206) or unknown/missing nodal status (n=256) are not included. HR, hormone receptor; [N+(mic,1-3)], micrometastases up to three positive nodes.
