# Supplementary material for: Breast-cancer-specific mortality in patients treated based on the 21-gene assay: a SEER population-based study
Source: NPJ Breast Cancer. 2016 Jun 8;2:16017–. doi: 10.1038/npjbcancer.2016.17 (PMC5515329; doi:10.1038/npjbcancer.2016.17)
Supplement: Supplementary Table [file npjbcancer201617-s2.doc]

Supplemental Table 1. Five-year breast cancer-specific mortality (subgroup analyses).

| **Subgroup** | **RS Group** | **Node-negative (N=40,134)** | | | **Node-positive [N+(mic,1-3)] (N=4,691)** | | |
| --- | --- | --- | --- | --- | --- | --- | --- |
|  |  | **n (%)^1^** | **5-year BCSM (SE)** | **Log-rank  p-value** | **n (%)^1^** | **5-year BCSM (SE)** | **Log-rank**  **p-value** |
| Age, years | | | | | | | |
| <40 | <18 | 682 (1.7) | 0 (0) | 0.0011 | 82 (1.7) | 1.6 (1.60) | 0.0354 |
|  | 18-30 | 637 (1.6) | 0.2 (0.22) |  | 62 (1.3) | 0 (0) |  |
|  | ≥31 | 161 (0.4) | 5.2 (3.23) |  | 21 (0.4) | 15.1 (9.86) |  |
| 40-49 | <18 | 5185 (12.9) | 0.2 (0.09) | <.0001 | 507 (10.8) | 1.0 (0.99) | <.0001 |
|  | 18-30 | 3550 (8.8) | 1.1 (0.26) |  | 285 (6.1) | 0.5 (0.54) |  |
|  | ≥31 | 615 (1.5) | 2.0 (0.83) |  | 61 (1.3) | 12.0 (7.34) |  |
| 50-59 | <18 | 6799 (16.9) | 0.2 (0.07) | <.0001 | 757 (16.1) | 1.4 (0.81) | 0.1125 |
|  | 18-30 | 4924 (12.3) | 1.3 (0.25) |  | 515 (11.0) | 4.6 (1.84 |  |
|  | ≥31 | 1021 (2.5) | 3.1 (0.82) |  | 103 (2.2) | 9.5 (9.06) |  |
| 60-69 | <18 | 6471 (16.1) | 0.6 (0.14) | <.0001 | 827 (17.6) | 0.3 (0.19) | <.0001 |
|  | 18-30 | 4438 (11.1) | 1.3 (0.25) |  | 501 (10.7) | 2.0 (1.08) |  |
|  | ≥31 | 1004 (2.5) | 5.2 (1.02) |  | 90 (1.9) | 15.5 (7.62) |  |
| 70-79 | <18 | 2360 (5.9) | 1.2 (0.43) | <.0001 | 464 (9.9) | 1.6 (0.92) | 0.0007 |
|  | 18-30 | 1439 (3.6) | 2.3 (0.58) |  | 267 (5.7) | 0.8 (0.53) |  |
|  | ≥31 | 374 (0.9) | 10.4 (3.07) |  | 47 (1.0) | 15.4 (7.38) |  |
| ≥ 80 | <18 | 263 (0.7) | 0.4 (0.39) | 0.0001 | 57 (1.2) | 0 (0) | 0.0122 |
|  | 18-30 | 164 (0.4) | 7.3 (2.53) |  | 39 (0.8) | 3.2 (3.17) |  |
|  | ≥31 | 47 (0.1) | 21.6 (8.77) |  | 6 (0.1) | 33.3 (19.25) |  |
| Tumor grade | | | | | | | |
| Well | <18 | 7521 (19.3) | 0.2 (0.09) | <.0001 | 938 (20.4) | 1.1 (0.63) | 0.8481 |
|  | 18-30 | 3534 (9.1) | 0.7 (0.18) |  | 380 (8.3) | 0.9 (0.84) |  |
|  | ≥31 | 153 (0.4) | 2.4 (1.68) |  | 15 (0.3) | 0 (0) |  |
| Moderate | <18 | 11681 (30.0) | 0.4 (0.09) | <.0001 | 1456 (31.7) | 1.2 (0.53) | 0.0006 |
|  | 18-30 | 8174 (21.0) | 1.1 (0.17) |  | 932 (20.3) | 2.7 (1.04) |  |
|  | ≥31 | 1180 (3.0) | 4.3 (0.95) |  | 129 (2.8) | 6.9 (3.08) |  |
| Poor | <18 | 1860 (4.8) | 0.5 (0.25) | <.0001 | 239 (5.2) | 0 (0) | <.0001 |
|  | 18-30 | 3017 (7.7) | 2.7 (0.48) |  | 324 (7.1) | 3.5 (1.74) |  |
|  | ≥31 | 1827 (4.7) | 4.8 (0.76) |  | 179 (3.9) | 23.7 (7.59) |  |
| Tumor size (mm) | | | | | | | |
| ≤5 | <18 | 756 (1.9) | 0.4 (0.27) | 0.3383 | 79 (1.7) | 4.2 (4.08) | 0.0005 |
|  | 18-30 | 446 (1.1) | 1.3 (0.76) |  | 56 (1.2) | 0 (0) |  |
|  | ≥31 | 79 (0.2) | 1.9 (1.85) |  | 7 (0.1) | 33.3 (27.22) |  |
| >5 to 10 | <18 | 4900 (12.3) | 0.2 (0.09) | <.0001 | 423 (9.1) | 0 (0) | 0.1598 |
|  | 18-30 | 3356 (8.4) | 0.7 (0.22) |  | 243 (5.2) | 1.6 (1.24) |  |
|  | ≥31 | 468 (1.2) | 2.3 (1.06) |  | 23 (0.5) | 0 (0) |  |
| >10 to 20 | <18 | 11462 (28.8) | 0.3 (0.07) | <.0001 | 1368 (29.3) | 0.8 (0.49) | <.0001 |
|  | 18-30 | 8020 (20.1) | 1.1 (0.18) |  | 819 (17.5) | 1.9 (0.72) |  |
|  | ≥31 | 1618 (4.1) | 3.2 (0.66) |  | 145 (3.1) | 7.5 (3.45) |  |
| >20 to 40 | <18 | 4002 (10.0) | 0.8 (0.21) | <.0001 | 684 (14.7) | 0.7 (0.54) | <.0001 |
|  | 18-30 | 2899 (7.3) | 2.5 (0.43) |  | 453 (9.7) | 3.8 (2.02) |  |
|  | ≥31 | 946 (2.4) | 8.2 (1.46) |  | 134 (2.9) | 25.1 (10.47) |  |
| >40 | <18 | 500 (1.3) | 2.4 (1.55) | 0.0054 | 129 (2.8) | 3.2 (1.94) | 0.1993 |
|  | 18-30 | 322 (0.8) | 6.0 (2.23) |  | 86 (1.8) | 4.3 (4.16) |  |
|  | ≥31 | 92 (0.2) | 9.2 (4.36) |  | 18 (0.4) | 32.1 (20.73) |  |
| Race | | | | | | | |
| White | <18 | 18372 (46.1) | 0.4 (0.07) | <.0001 | 2322 (49.7) | 1.0 (0.39) | <.0001 |
|  | 18-30 | 12710 (31.9) | 1.4 (0.15) |  | 1433 (30.7) | 2.4 (0.75) |  |
|  | ≥31 | 2602 (6.5) | 4.4 (0.61) |  | 266 (5.7) | 15.9 (4.21) |  |
| Black | <18 | 1472 (3.7) | 0.8 (0.28) | <.0001 | 178 (3.8) | 0.6 (0.56) | 0.4117 |
|  | 18-30 | 1099 (2.8) | 1.8 (0.58) |  | 119 (2.5) | 1.1 (1.12) |  |
|  | ≥31 | 319 (0.8) | 5.7 (2.17) |  | 31 (0.7) | 8.7 (8.31) |  |
| Other | <18 | 1796 (4.5) | 0.2 (0.15) | <.0001 | 183 (3.9) | 1.5 (1.53) | 0.8427 |
|  | 18-30 | 1247 (3.1) | 0.8 (0.36) |  | 110 (2.4) | 0.9 (0.92) |  |
|  | ≥31 | 278 (0.7) | 4.7 (1.89) |  | 27 (0.6) | 0 (0) |  |
| Socioeconomic quintile | | | | | | | |
| Lowest | <18 | 2418 (6.1) | 0.7 (0.24) | <.0001 | 309 (6.7) | 1.5 (1.22) | 0.3578 |
|  | 18-30 | 1561 (4.0) | 2.2 (0.55) |  | 162 (3.5) | 1.2 (1.16) |  |
|  | ≥31 | 438 (1.1) | 6.1 (1.86) |  | 38 (0.8) | 10.5 (9.96) |  |
| Second lowest | <18 | 3199 (8.1) | 0.5 (0.16) | <.0001 | 393 (8.5) | 1.6 (1.34) | <.0001 |
|  | 18-30 | 2136 (5.4) | 1.2 (0.36) |  | 259 (5.6) | 0 (0) |  |
|  | ≥31 | 499 (1.3) | 5.6 (1.57) |  | 51 (1.1) | 24.8 (14.78) |  |
| Middle | <18 | 4076 (10.3) | 0.4 (0.16) | <.0001 | 468 (10.1) | 1.5 (0.90) | <.0001 |
|  | 18-30 | 2729 (6.9) | 1.5 (0.35) |  | 289 (6.3) | 2.0 (1.32) |  |
|  | ≥31 | 607 (1.5) | 5.4 (1.34) |  | 59 (1.3) | 11.9 (5.11) |  |
| Second highest | <18 | 4953 (12.5) | 0.4 (0.13) | <.0001 | 640 (13.9) | 1.1 (0.95) | <.0001 |
|  | 18-30 | 3530 (8.9) | 1.1 (0.26) |  | 406 (8.8) | 1.3 (1.08) |  |
|  | ≥31 | 715 (1.8) | 4.0 (1.21) |  | 73 (1.6) | 23.5 (10.82) |  |
| Highest | <18 | 6770 (17.1) | 0.3 (0.10) | <.0001 | 845 (18.3) | 0.4 (0.26) | 0.0029 |
|  | 18-30 | 4947 (12.5) | 1.3 (0.24) |  | 520 (11.3) | 3.6 (1.47) |  |
|  | ≥31 | 919 (2.3) | 3.1 (0.84) |  | 100 (2.2) | 9.4 (5.28) |  |

^1^Among patients in the cohort with nonmissing information.

BCSM, breast cancer-specific mortality; [N+(mic,1-3)], node-positive (micrometastases and up to three positive nodes); RS, Recurrence Score result; SE, standard error.
