# Supplementary material for: Breast-cancer-specific mortality in patients treated based on the 21-gene assay: a SEER population-based study
Source: NPJ Breast Cancer. 2016 Jun 8;2:16017–. doi: 10.1038/npjbcancer.2016.17 (PMC5515329; doi:10.1038/npjbcancer.2016.17)
Supplement: Supplementary Figure [file npjbcancer201617-s3.ppt]

## Slide 1
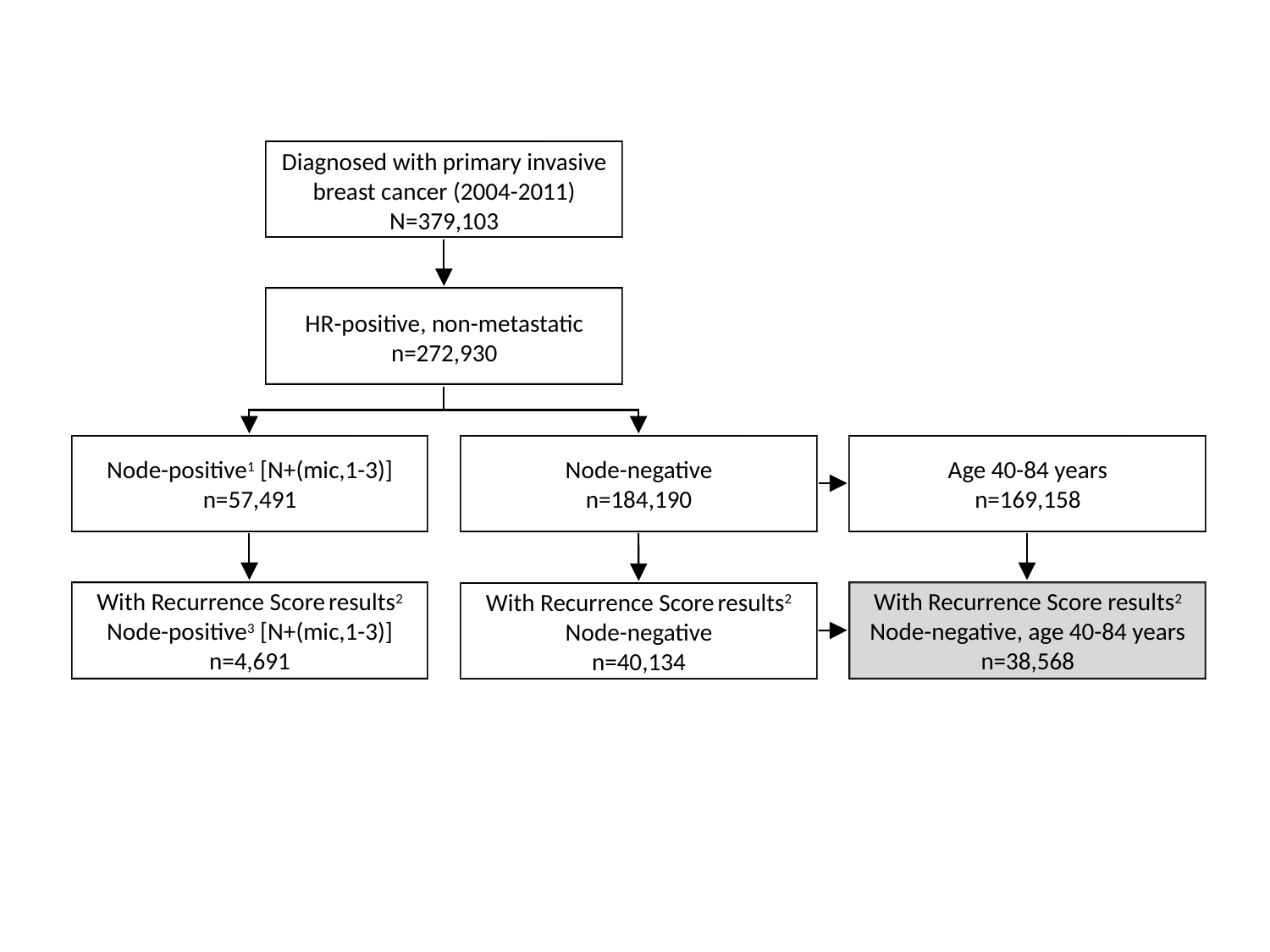

Diagnosed with primary invasive breast cancer (2004-2011)
N=379,103
HR-positive, non-metastatic
n=272,930
Node-positive1 [N+(mic,1-3)]
n=57,491
Node-negative
n=184,190
Age 40-84 years
n=169,158
With Recurrence Score results2
Node-positive3 [N+(mic,1-3)]
n=4,691
With Recurrence Score results2
Node-negative, age 40-84 years
n=38,568
With Recurrence Score results2
Node-negative
n=40,134
